# Study of MIC of silver and zinc oxide nanoparticles, strong and cost-effective antibacterial against biofilm-producing *Acinetobacter baumannii* in Shiraz, Southwest of Iran

**DOI:** 10.1186/s12879-024-09471-1

**Published:** 2024-06-17

**Authors:** Farshad Kakian, Nawal Arasteh, Esmaeil Mirzaei, Mohammad Motamedifar

**Affiliations:** 1 Department of Bacteriology and Virology, School of Medicine, Shiraz University of Medical Sciences, Shiraz, Iran; 2 Department of Medical Nanotechnology, School of Advanced Medical Sciences and Technologies, Shiraz University of Medical Sciences, Shiraz, Iran; 3 HIV/AIDS Research Center, Institute of Health, Shiraz University of Medical Sciences, Shiraz, Iran

**Keywords:** *Acinetobacter baumannii*, Polymerase chain reaction, Nanoparticles

## Abstract

**Background:**

*Acinetobacter baumannii* resistant strains lead to increased mortality, treatment costs, and an increase in the length of hospitalization. Nowadays, nanoparticles are considered a substitute for antibiotics. This study aimed to determine the MIC of Silver (Ag) and Zinc Oxide (ZnO) Nanoparticles (NPs) on Biofilm-Producing *Acinetobacter baumannii* and determine the relationship between MIC and frequency of efflux pump genes in cutaneous specimens in Shiraz, Southwest Iran in 2021–2022.

**Methods:**

In this study, specimens were collected from April 2021 to June 2022 at Namazi and Faqihi Hospitals in Shiraz. Investigation of biofilm production in multidrug resistance (MDR) isolates was done by the microtiter plate method. Synthesized nanoparticles were characterized by UV–vis spectrum, X-ray diffraction (XRD), and electron microscopy. The MIC of AgNPs and ZnONPs for isolates was done using the method described in the CLSI guideline (2018). The antibacterial effect of MIC of NPs on inanimate objects was done by colony counts. The prevalence of efflux pump genes (*adeR*, *adeC*, *adeA*, *abeM*, *adeK*, *adeI*) was also investigated by PCR technique.

**Results:**

The highest ceftriaxone resistance (68%) and lowest colistin resistance (7%) were identified. 57% of isolates were MDR. In addition, 71.9% could produce biofilm and 28.1% of isolates could not produce biofilm. The average size of AgNPs and ZnONPs in the present study is 48 and < 70 nm, respectively. The nanoparticles were spherical. The MIC and the MBC of the ZnONPs were in the range of 125 to 250 µg/mL respectively. Also, for AgNPs, the MIC and the MBC were in the range of 62.5 to 250 µg/ml, respectively. *AbeM* gene had the highest frequency and the *AdeK* gene had the lowest frequency. Statistical analysis showed that there is a relationship between the frequency of *adeA*, *adeC*, and *adeM* genes with the MIC of AgNPs and ZnONPs.

**Conclusion:**

According to the results of the present study, inanimate objects such as scalpels in contact with AgNPs (6000 µg/ml for 240 min) or ZnONPs (5000 µg/ml for 120 min) can be free of biofilm producing *Acinetobacter baumannii*  with efflux pump genes.

## Background

*Acinetobacter baumannii* is a bacteria related to nosocomial-acquired infection, principally in the Intensive Care Units (ICU) that contain bacteremia, meningitis, urinary tract infection, pneumonia and wound infections [1, 2]. Virulence factors such as biofilm are factors that contribute to the spread of pathogenic bacteria [3]. Reports have shown an increase in multi, extensive and pan- drug resistance (MDR, XDR, and PDR) in *Acinetobacter baumannii*, some of which are even resistant to colistin [1, 4]. Studies have shown virulence factors, such as biofilm genes, play a significant role in the high level of treatment failure between wound infections produced by *A. baumannii* isolates [5]. Evidence suggests that the ability of *A. baumannii* to form biofilms increases antibiotic resistance/tolerance as well as persistence in hospital settings [6]. The antibacterial mechanisms of nanoparticles (NPs) are not completely understood, but accepted mechanisms include oxidative stress induction, metal ion release, non-oxidative mechanisms, cell membrane damage, DNA damage, and free radical generation [7]. Moreover, the antimicrobial properties of NPs depend on different factors such as their synthesis method, NPs size, morphology, etc [8]. Therefore, considering the importance of human skin and the need to disinfect hospital surfaces and medical equipment and tools to remove resistant bacteria, this study is aimed at determining efflux pump genes and the effect of silver nanoparticles and zinc oxide on Biofilm-producing *Acinetobacter baumannii* isolates from skin samples (Wound pus) in Shiraz, Southwest of Iran.

## Methods

### Synthesis of nanoparticles

A chemical reduction method was used to synthesize AgNPs. Ascorbic acid was used as a reducing agent and sodium citrate as a stabilizing agent. First, ascorbic acid was dissolved in 64 ml of distilled water and then 49.52 mg of sodium citrate was added to it. Then we adjusted the pH to 10.5 with NaOH. In the next step, we added silver salt (AgNo3) dissolved in distilled water (10 mg in 64 ul) to the solution. We allowed AgNPs to form on the hot plate for 30 min at a temperature of 30 ^C^. In the last step, centrifugation was performed at 10,000 rpm for 40 min and falcon sediment was used [9]. In this study, ZnONPs powder was purchased from the Burhan nanoscale innovators company of the Ferdowsi University of Mashhad and used. These nanoparticles have been characterized using UV-Vis, XRD, and transmission electron microscopy (TEM) methods. In this study, ZnONPs with spherical shape and less than 70 nm size were used. This study was approved by the Ethics Committee of Shiraz University of Medical Sciences (Ethics code: *IR.SUMS.REC.1400.876)*.

### UV–Vis spectroscopy

The AgNPs were characterized by Unico UV-2100 Spectrophotometer. The scanning range for the samples was 300–500 nm. The double distilled water was used as a blank reference [10].

### X‑ray diffraction analysis

The X-ray diffraction data for AgNPs were obtained by X-ray diffractometer (ASENWARE, AW-XDM300 with Cu Kα radiation sources (λ = 0.154 nm) by operating voltage 18 kV and the range of diffraction angle were set as 10°-80°) [10].

### Scanning electron microscopy

The morphology and size of the Ag particle were determined using a scanning electron microscope (SEM), (X13, Philips, America Leo 1430vp) [10].

### Isolation of *A. baumannii*

Isolates of *A. baumannii* were provided by the Clinical Laboratory of the Faghihi and Namazi Hospital, Shiraz, Iran. All isolates were collected from wound cultures (Wound pus). Isolates were cultured on EMB and blood agar and after 24 h incubation at 24 °C the colonies were observed.

#### Identification of *A. baumannii*

Single colonies were subjected to Gram staining for morphological characterization. Colonies of Gram-negative rod-shaped coccobacillus cells were used to confirm with urease, oxidase, OF (Oxidative-fermentation), citrate, catalase and TSI tests. Genotypic confirmation of the isolates was done by PCR for bla*oxa-51* gene. A fragment (353 bp) of *blaoxa-51* gene was amplified by specific primer pairs which were reported previously, Forward, 5′-TAATGCTTTGATCGGCCTTG − 3′, and Reverse, 5′-TGGATTGCACTTCATCTTGG − 3′ [11].

### Antimicrobial susceptibility tests

#### Antibiotic susceptibility testing

The antibiogram test was done by Kirby-Bauer method for gentamicin (30 mg), colistin (30 µg), imipenem (10 µg), meropenem (10 µg), ciprofloxacin (5 µg), amikacin (30 mg), cefepime (30 mg), Trimethoprim–sulfamethoxazole (30 mg), Ceftazidime (30 mg), Ceftriaxone (30 mg), and Tetracycline (10 µg) (Himedia, India), according to standard guideline of CLSI. *E. coli* ATCC 25,922 and *Pseudomonas aeruginosa* ATCC 27,853, were used as the control. Results were recorded as sensitive(S), intermediate (I) and resistant(R) on the basis of Clinical and Laboratory Standard Institute (CLSI). Isolates with multidrug resistance (MDR) were also identified. Biofilm production was investigated in isolates with multidrug resistance.

#### Biofilm assay

The micro titer plate technique was conducted as before described. Briefly, 200 µl of bacterial suspension grown in trypticase soy broth (TSB), supplemented with 1% glucose and diluted. A total of 1:100 was poured into the wells of sterile flat-bottomed 96-well polystyrene tissue culture plates (200 µl of TSB supplemented with 1% glucose was used as the negative control) and incubated 24 h at 37 °C. All tests were performed in triplicate. Washing was then performed three times for each well with sterile phosphate-buffered saline (PBS; pH 7.2). After that, the fixation step was done by air drying. Subsequently, the adherent biofilm layer was stained by crystal violet for 15 min at room temperature. This was followed by the washing steps. Then the plates were air dried and resolubilized with ethanol (95%) for 30 min. Finally, the optical density (OD) of each well was measured at 570 nm, and average OD value of negative controls and samples was calculated. Interpretation of results was described as follows [12]:

ODs ≤ ODc = no biofilm producer; ODc ≤ ODs ≤ 2 × ODc = weak biofilm producer; 2 × ODc ≤ ODs ≤ 4× ODc = moderate biofilm producer; 4 × ODc < ODs = strong biofilm producer (ODc = OD control negative, ODs = OD samples). *Pseudomonas aeruginosa* ATCC 27,853 was used as the biofilm producer control strain.

#### MIC of AgNPs and ZnONPs

Susceptibility of biofilm-producing *Acinetobacter baumannii* isolates against AgNPs and ZnONPs were evaluated by microbroth dilution method which was developed based on Clinical and Laboratory Standard Institute (CLSI) approved techniques. 100 µl of MHB was added to the 12 to 1 well. Then 100 µl of nanoparticle stock solution (1000 ug/mL for AgNPs and 2000 ug/mL for ZnONPs) was poured into well 12 and serial dilution was prepared from well 12 to 3. Then, 5 µl of bacteria whit a concentration of 10^7^ CFU/mL were added to all the wells except the negative control well. Finally, the microplate was incubated at 37 ^C^ for 24 h. The first dilution in which no growth was observed was considered MIC [13].

#### MBC of AgNPs and ZnONPs

When the MIC determination for each NPs, 50 ul from all the wells which showed no visible bacterial growth were inoculated on MH agar plates and incubated for 24 h at 37 ^C^. When 99.9% of the bacterial population is killed at the lowest concentration of a nanoparticle agent, it is called an MBC endpoint [13].

#### Antibacterial effect on inanimate objects

Nanoparticles were prepared in sterile distilled water. For this aim we used ZnONPs (4000, 5000 and 6000 µg/mL) and AgNPs (4000, 5000 and 6000 µg/ml) concentrations. Medical scalpels, which were contaminated with *Acinetobacter baumannii*, were studied for decontamination with these concentrations of nanoparticles. They were exposed to nanoparticles at room temperature for 15, 30, 60, 120 and 240 min. Then colony forming unites (CFU) was done by culturing in blood agar and EMB at 37^c^ for 24 h. Lack of growth (99.9%) was the criterion for us.

#### DNA extraction and polymerase chain reaction (PCR)

The DNA extracted by boiling method. First, 3–5 colonies were inoculated into a microtube containing 1000 µl of Tris buffer. The next step was heating (hot plate for 15 min at 95 °C) and then centrifugation (14,000 rpm for 10 min). The supernatant was used as DNA [14]. The detection of efflux pump genes was performed using PCR for MDR isolates. Primers for this study are shown in Table 1 [15, 16]. The frequency of these genes as well as the statistical relationship between their prevalence and the concentration of nanoparticles were investigated.

**Table 1:** Primers used for amplification of the efflux pump genes examined

Gene	Primer	Size (bp)	Tm
*adeR*	F: ATGTTTGATCATTCTTTTTCTTTTG R: TTAATTAACATTTGAAATATG	687	47
*adeC*	F: TACACATGCGCATATTGGTG R: CGTAAAATAACTATCCACTCC	117	52
*adeA*	F: ATCGCTAACAAAGGCTTGAA R: CGCCCCCTCAGCTATAGAA	159	52
*adeI*	F: CAAATGCAAATGTAGATCTTGG R: AAACTGCCTTTACTTAGTTG	210	56
*adeK*	F: TTGATAGTTACTTGACTGTTC R: GGTTGGTGAACCACTGTATC	162	50
*abeM*	F: TATTACTTACCTTGCAACGCAG R: GTGGTTGCAATCATGATGCCA	283	55

### Statistical analysis

Totally antimicrobial tests were performed in triplicate and data were statistically analyzed by software Statistical Package for the Social Sciences (IBM, SPSS Statistics, and Version 25). Independent sample T-test was used for analysis. *P*-value less than 0.05 was considered as significant.

## Results

### UV–Vis spectroscopy

**Fig. 1:**
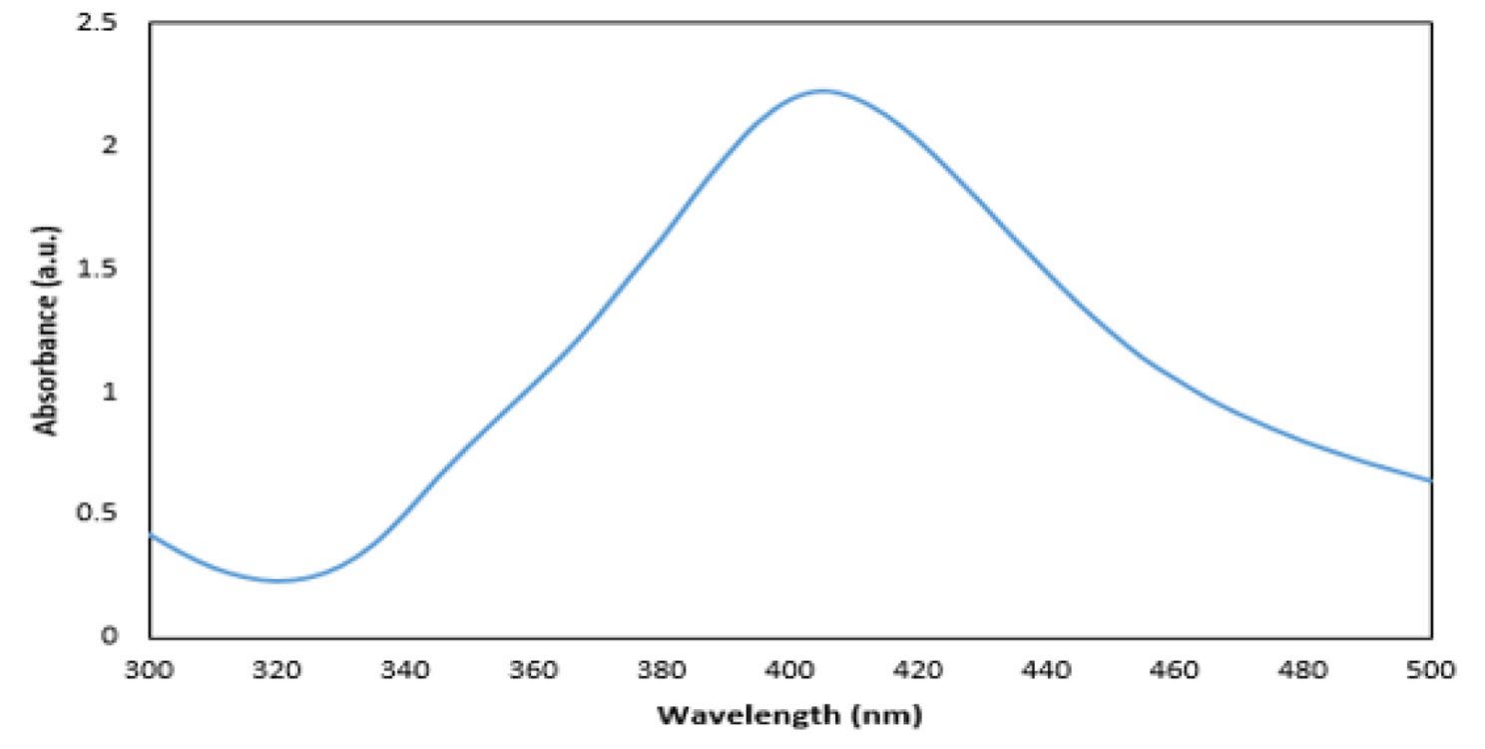
UV–vis spectra of AgNPs

Figure 1 shows the UV–vis spectra of the prepared AgNPs. A strong peak specific for the synthesis of AgNPs was obtained at 410 nm which was specific to AgNPs.

### X‑ray diffraction analysis

**Fig. 2:**
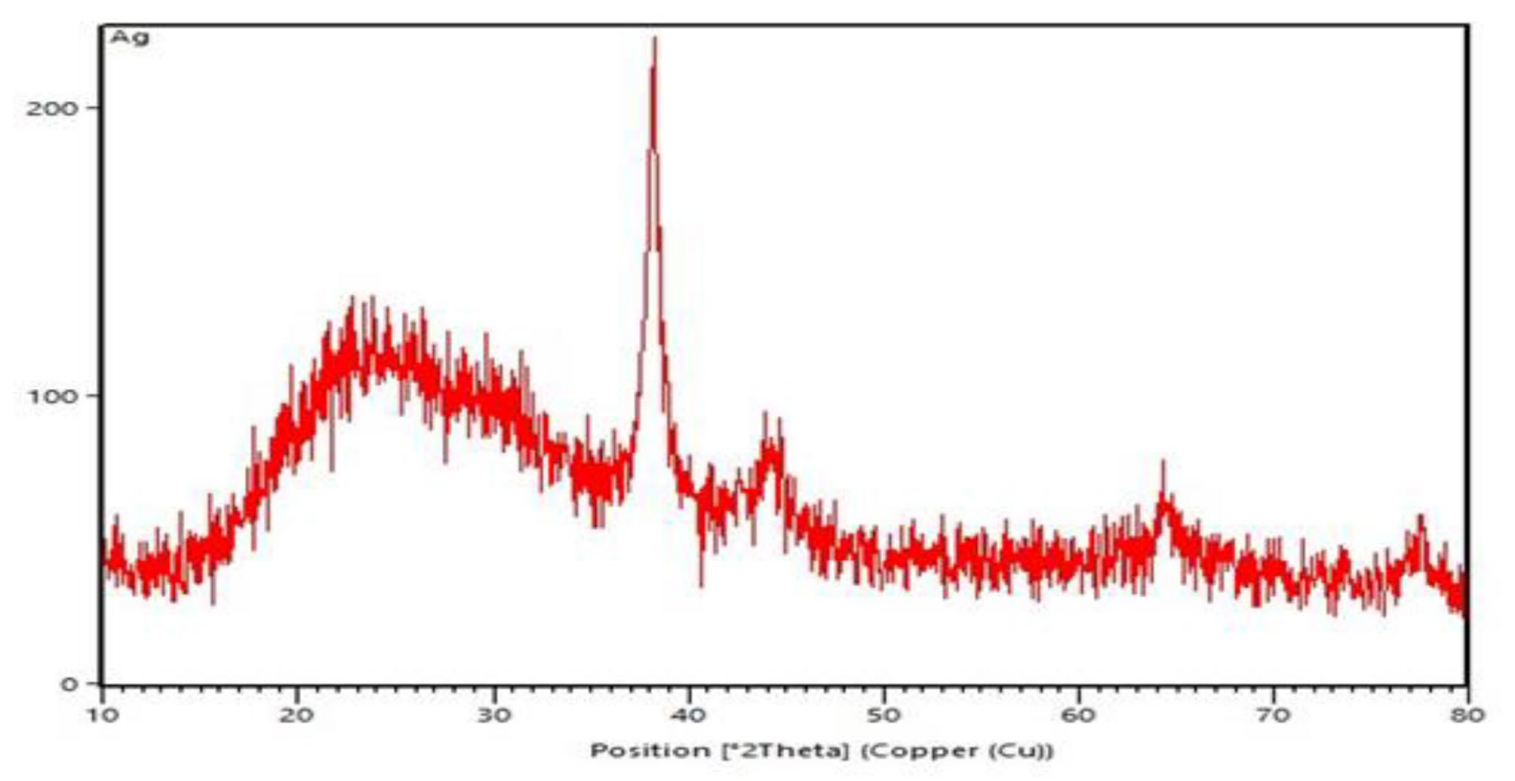
XRD spectra of AgNPs

The crystalline nature of the AgNPs was performed by XRD. As can be seen in Fig. 2, characteristic diffraction peak of AgNPs was appeared at ~ 38°, 44° and 63° of 2-theta.

### Electron microscopy

**Fig. 3:**
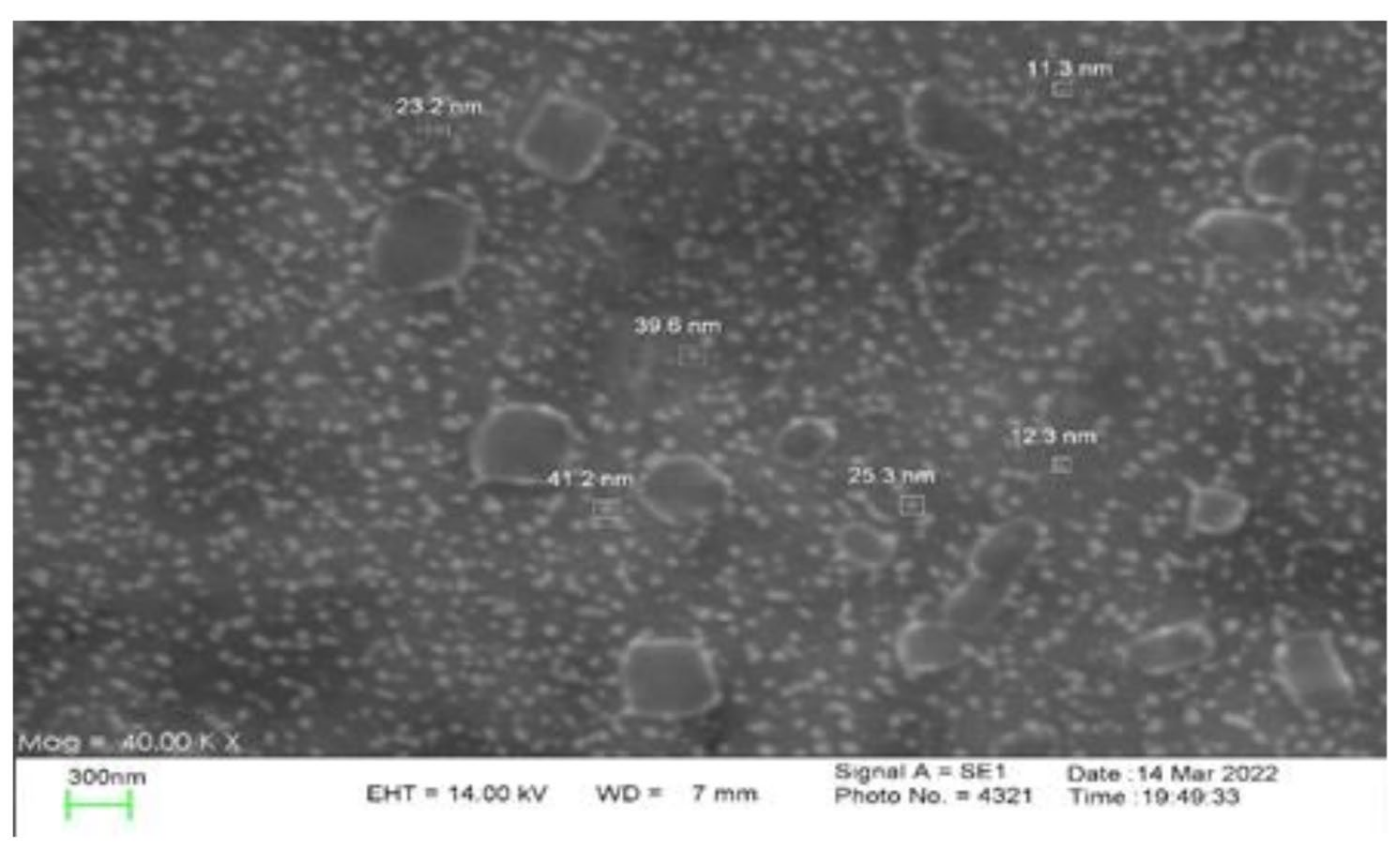
SEM micrographs of the prepared AgNPs

Figure 3 depicts the SEM micrographs of the AgNPs. The Characterization of AgNPs was confirmed using SEM (X13, Philips, America Leo 1430vp) that the average size of the nanoparticles is about 48 nm and they are spherical. In this study, ZnONPs with spherical shape and less than 70 nm size used.

### Isolation of A. Baumannii

Out of 122 isolates identified by biochemical tests, 100 isolates were finally confirmed by PCR. Out of 100 studied isolates, 57% were from men and 43% from women (1–94 years old). Most isolates have been isolated from the intensive care unit (47%). No significant relationship was observed between wards, age and antibiotic resistance (*p* < 0.05). Oxa-51 (353 bp) gene was the genotypic confirmation of the isolates.

#### Antibiotics susceptibility test

Highest Ceftriaxone resistance (68%) and lowest Colistin resistance (7%) were identified. 57% isolates were MDR. Resistance to Piperacillin /Tazobactam, Meropenem and Imipenem was 62%. Also, 26% resistance to Tetracycline was observed.

#### Biofilm assay

In our study, 71.9% could produce biofilm and 28.1% of isolates could not produce biofilm. 56.1% isolates were strong, 8.7% were medium and 7.1% were weak biofilm producing.

#### MIC and MBC of AgNPs and ZnONPs

The MIC and the MBC of the ZnONPs were in the range of 125 µg/ml to 250 µg/mL, respectively. Also, for AgNPs, the MIC and the MBC were in the range of 62.5 µg/mL to 250 µg/mL, respectively. In the present study, MBC for AgNPs and ZnONPs was the same concentration that was obtained for the MIC. The MIC and MBC showed that *A. baumannii* is more sensitive to AgNPs than to ZnONPs. Statistical analysis showed that there is a direct relationship between the strength of biofilm production and the MIC of nanoparticles, and isolates with strong biofilm had higher MIC (*p* = 0.000). Also, no significant relationship was observed between antibiotic resistance and the MIC.

#### Antibacterial effect on inanimate objects

The results for ZnONPs showed that no growth was observed after 120 min at a concentration of 5000 µg/ml ZnONPs. However, at a concentration of 4000 µg/ml, growth was observed after 240 min. Also, AgNPs at a 6000 µg/ml (240 min) were able to inhibit bacteria, and growth was observed at a concentration of 6000 for 120 min.

#### Polymerase chain reaction (PCR)

*abeM* gene had the highest frequency and the *adeK* gene had the lowest frequency (Table 2). Statistical analysis showed that there is a relationship between the frequency of *adeA*, *adeC* and *adeM* genes and the MIC level of NPs. As you can see in Table 3, these genes were more prevalent at higher MIC of nanoparticles, and with decreasing MIC, their prevalence decreased and it is statistically significant.

**Table 2:** Frequency of studied genes

Gene	Positive (%)	Negative (%)
*abeM*	54 (94.73%)	3 (5.26%)
*adeA*	51 (89.47%)	6 (10.52%)
*adeC*	46 (80.70%)	11 (19.29%)
*adeI*	30 (52.63%)	27 (47.36%)
*adeR*	28 (49.12%)	29 (50.87%)
*adeK*	8 (14.03%)	49 (85.96%)

**Table 3:** The relationship between the frequency of efflux pump genes with the MIC of NPs (%)

Gene	AgNP MIC = 250 (*N* = 15)	AgNP MIC = 125 (*N* = 24)	AgNP MIC = 62.5 (*N* = 2)	*P*- Value	ZnO NP MIC = 250 (*N* = 34)	ZnO NP MIC = 125 (*N* = 7)	*P*- Value
*adeA*	15 (100%)	18 (75%)	0 (0%)	0.000	32 (94.11%)	3 (42.85)	0.000
*adeR*	2 (13.33%)	12 (50%)	0 (0%)	0.684	10 (29.41%)	5 (71.42%)	0.079
*adeK*	1 (6.66%)	6 (25%)	0 (0%)	0.752	2 (5.88%)	1 (14.28%)	0.058
*adeI*	9 (60%)	19 (79.16%)	1 (50%)	0.544	12 (35.29%)	5 (71.42%)	0.344
*adeC*	15 (100%)	18 (75%)	0 (0%)	0.000	33 (97.05%)	2 (28.57%)	0.000
*abeM*	15 (100%)	20 (83.33%)	1 (50%)	0.000	34 (100%)	3 (42.85)	0.000

## Discussion

Antibiotic-resistant isolates in medical centers cause an increase in mortality and treatment costs. Among the bacterial agents responsible for hospital infections, *Acinetobacter* is receiving a lot of attention due to its high resistance [17]. Today, nanoparticles are considered as an important alternative to antibiotic therapy [18]. Previous studies show that the synthesis methods of nanoparticles are different and chemical synthesis is one of the standard methods. Also, in other studies, like the present study, it has been shown that silver and zinc oxide nanoparticles are a suitable treatment option for multi-drug resistant *Acinetobacter baumannii* isolates [19, 20]. The data obtained for the characterization of nanoparticles (UV-vis, XRD and Electron microscopy), as in previous studies, are within the appropriate range and indicate the correct synthesis of nanoparticles. These data strongly confirm that the nanoparticles are correctly formed [21–24]. In this study, the lowest resistance was observed in colistin, tetracycline and gentamicin/amikacin, respectively. In terms of biofilm production power, like other studies, all the isolates were not able to produce biofilm [25–27]. In the investigations carried out in Iran by Shokrollahiet al. and the study conducted by Łysakowska et al. in Poland, contrary to the present study, they showed that there is a significant relationship between MIC of nanoparticles and antibiotic resistance [28, 29]. In the study conducted by Hendiani et al. in Iran, like the study conducted in other parts of the world, in line with the present study, they concluded that there is a significant relationship between the power of biofilm production and the MIC of Ag and ZnO NPs [30–32].

The prevalence of investigated efflux pump genes has been different in different studies. However, in the study of Angoti et al., as in the present study, the frequency of *adeM* (98.3%) and *adeA* (88.5%) genes has been reported more [16]. In the study of Ranjbar et al. and also in the study of Jassim et al., they found the highest frequency to be related to the *adeA* and *adeC* gene [33, 34]. In this study, it was found that the genes that are more abundant are related to isolates whose growth was inhibited by a higher concentration of nanoparticles, and the abundance of these genes was related to the concentration of nanoparticles. This importance is in line with the studies conducted in other regions and it can be assumed that the bacteria increase these genes to deal with the higher concentration of nanoparticles or take steps to deal with the nanoparticle [35–37]. Based on the evidence obtained from this study, colistin has good in vitro activity against *A. baumannii* isolates. Therefore, this antibiotic, like other studies [38], can be used as a last treatment option for patients with MDR/XDR *A. baumannii*. Therefore, due to the high resistance to other antibiotics, the optimal use of polymyxin is necessary and they can be prescribed in combination with other antibiotics in order to greater effectiveness. The findings of this study can lead to the production of nanoparticles with an antimicrobial purpose, and economically, they can be cost-effective compared to antibiotic therapy. Nanoparticles can be synthesized from plant extracts. They are easy to synthesize and are more environmentally sustainable. In addition, they can be produced on a large scale [39, 40]. Therefore, they are more economical than antibiotics.

## Conclusion

ZnONPs and AgNPs were considered a new and promising nanoparticle for bacterial infections. In recent years, many studies have been conducted to find the mechanisms of action of these nanoparticles. The results of this study have a suggestion and a message that silver and zinc oxide nanoparticles can remove biofilm producing *Acinetobacter baumannii* with efflux pump genes from inanimate objects.

## Data Availability

The datasets used and/or analyzed during the current study are available from the corresponding author on reasonable request.

## Abbreviations

Ag: Silver
ZnO: Zinc Oxide
NPs: Nanoparticles
XRD: X-ray diffraction
CLSI: Clinical & Laboratory Standards Institute
CFU: Colony-forming units
PCR: Polymerase chain reaction
SEM: scanning electron microscopy
MIC: Minimum inhibitory concentration
MDR: multidrug resistance
